# Antibacterial Potential and Phytochemical Composition in Subcritical Water Extraction of Lempoyang (*Zingiber zerumbet*)

**DOI:** 10.21315/tlsr2025.36.1.9

**Published:** 2025-03-30

**Authors:** Nurul ‘Uyun Ahmad, Mariam Firdhaus Mad Nordin, Norrashidah Mokhtar, Tan Ka Liong, Izzati Mohamad Abdul Wahab, Muhamad Ali Muhammad Yuzir, Mardawani Mohamad

**Affiliations:** 1 School of Chemical Engineering, College of Engineering, Universiti Teknologi MARA, Cawangan Terengganu, Kampus Bukit Besi, 23200 Bukit Besi, Dungun, Terengganu, Malaysia; 2 Department of Chemical and Environmental Engineering, Malaysia-Japan International Institute of Technology, Universiti Teknologi Malaysia, Jalan Sultan Yahya Petra, 54100 Kuala Lumpur, Malaysia; 3 AM Zaideen Ventures Sdn Bhd, 35E-G-05, Jalan Wangsa Delima 5, KLSC 2, Seksyen 5 Wangsa Maju, 53300 Kuala Lumpur, Malaysia; 4 Faculty of Medicine and Health Sciences, Universiti Sains Islam Malaysia, Persiaran Ilmu, Putra Nilai, 71800 Nilai, Negeri Sembilan, Malaysia; 5 Faculty of Bioengineering and Technology, Universiti Malaysia Kelantan, Jeli Campus, 17600 Jeli, Kelantan, Malaysia

**Keywords:** Antibacterial Activity, GCMS, Phytochemical, Subcritical Water Extraction, *Zingiber zerumbet*, Aktiviti Antibakteria, GCMS, Fitokimia, Pengekstrakan Air Subkritikal, *Zingiber zerumbet*

## Abstract

Lempoyang, scientifically known as *Zingiber zerumbet*, is a plant rich in potential medicinal properties due to its numerous active ingredients. The aim of this study was to investigate the phytochemical composition and antibacterial potential of crude extracts of lempoyang obtained by subcritical water extraction (SWE). Fresh rhizomes of lempoyang were extracted using the one-factor-at-a-time (OFAT) approach with different extraction times (5 min, 10 min, 15 min, 20 min and 25 min), while other extraction parameters were kept constant. The resulting crude extracts, characterised by gas chromatography-mass spectrometry (GCMS), contained 13 different constituents. Among these, 2,6,10-cycloundecatrien-1-one,2,6,9,9-tetramethyl-,(E,E,E)- or zerumbone consistently had the highest percentage area under the peak across all extraction times, ranging from 17.15% to 28.72% at retention times of 19.215 min to 19.270 min. Qualitative screening of these crude extracts revealed the presence of phenolics, alkaloids, flavonoids, terpenoids, saponins and tannins, indicating the rich phytochemical diversity of lempoyang. However, steroids and anthocyanins have not been detected. In terms of antibacterial activity, disc diffusion using the Kirby-Bauer method showed positive results for the 25-minute crude extract against *Escherichia coli*, yielding a zone of inhibition of 8.63 ± 0.36 mm at a concentration of 100 mg/mL/disc. None of the extracts were found to have detectable antibacterial activity against *Bacillus subtilis*, *Staphylococcus aureus*, *Pseudomonas aeruginosa*, *Salmonella choleraesuis* and *Serratia marcescens*. These results emphasise the potential of SWE for extracting valuable compounds from fresh lempoyang rhizomes. At the same time, they highlight that different extraction times influence the phytochemical profile and antibacterial activity of the crude extracts at higher extract concentrations.

## Abstract (fr)

**Abstrak:**

Lempoyang, yang dikenali secara saintifik sebagai *Zingiber zerumbet*, merupakan tumbuhan yang kaya dengan potensi sifat perubatan disebabkan oleh pelbagai bahan aktifnya. Tujuan kajian ini adalah untuk menyiasat komposisi fitokimia dan potensi antibakteria ekstrak lempoyang segar yang diperolehi melalui pengekstrakan air subgenting (SWE). Rizom segar lempoyang diekstrak menggunakan pendekatan satu faktor pada satu-satu masa (OFAT) dengan pelbagai masa pengekstrakan (5 min, 10 min, 15 min, 20 min dan 25 min) sementara parameter pengekstrakan lain dikekalkan. Ekstrak mentah yang dihasilkan yang dicirikan oleh *gas chromatography-mass spectrometry* (GCMS) mendedahkan 13 konstituen berbeza. Antara konsituen tersebut, 2,6,10-cycloundecatrien-1-one,2,6,9,9-tetramethyl-,(E,E,E)-, atau zerumbone menunjukkan peratus kawasan tertinggi di bawah puncak pada semua masa pengekstrakan, antara 17.15% hingga 28.72% pada masa penahanan bermula dari 19.215 min hingga 19.270 min. Penyaringan kualitatif ekstrak mentah ini mendedahkan kehadiran fenol, alkaloid, flavonoid, terpenoid, saponin dan tanin, menunjukkan kepelbagaian fitokimia yang kaya dalam lempoyang. Walau bagaimanapun, steroid dan antosianin, tidak dikesan. Dalam konteks aktiviti antibakteria, ujian penyebaran cakera Kirby-Bauer menunjukkan hasil positif bagi 25-minit ekstrak mentah terhadap *Escherichia coli*, memberikan zon perencatan sebanyak 8.63 ± 0.36 mm pada kepekatan 100 mg/mL/cakera. Walau bagaimanapun, tiada aktiviti antibakteria yang dapat dikesan terhadap *Bacillus subtilis*, *Staphylococcus aureus*, *Pseudomonas aeruginosa*, *Salmonella choleraesuis* dan *Serratia marcescens* dalam mana-mana ekstrak. Penemuan ini menekankan potensi SWE mengekstrak sebatian berharga daripada rizom lempoyang segar dan pada masa yang sama menunjukkan bahawa masa pengekstrakan yang berbeza mempengaruhi profil fitokimia dan aktiviti antibakteria ekstrak mentah pada kepekatan tinggi.

**Highlights**

- **Rich Phytochemical Composition:** Lempoyang (*Zingiber zerumbet*) crude extracts, obtained via subcritical water extraction (SWE), contain diverse phytochemicals, including phenolics, alkaloids, flavonoids, terpenoids, saponins and tannins, with zerumbone being the dominant compound (17.15%–28.72% of the total composition)
- **Antibacterial Potential:** The 25-minute crude extract exhibited antibacterial activity against E. coli with a zone of inhibition of 8.63 ± 0.36 mm at a concentration of 100 mg/mL, although no detectable activity was observed against other tested bacteria (*B. subtilis, S. aureus, P. aeruginosa, S. choleraesuis and S. marcescens*).
- **Extraction Time Influence:** The study highlights the significance of extraction time in determining the phytochemical profile and antibacterial effectiveness of lempoyang crude extracts, showcasing the utility of SWE for extracting bioactive compounds.

## INTRODUCTION

Lempoyang or *Zingiber zerumbet* from the Zingiberaceae family, offers significant health benefits, including antioxidant properties (Raj *et al*. 2022; Sidahmed *et al*. 2015; Tian *et al*. 2020), antibacterial effects (Huong *et al*. 2020; Raj *et al*. 2022; Tian *et al*. 2020), and anti-cancer properties (Norfazlina *et al*. 2014; Sam *et al*. 2019; Sithara *et al*. 2018). Traditionally used as a culinary spice, consumed as an herbal tea, and employed as a remedy for mild fever, diarrhoea, loss of appetite and other ailments. Lempoyang has been recognised for its diverse applications. Literature searches have unveiled various phytochemical compounds within the lempoyang rhizome. Zerumbone, found abundantly in the rhizome, stands out as the main bioactive compound renowned for its multifunctionality and remarkable medicinal properties (Ibáñez *et al*. 2023; Tan *et al*. 2018).

A crucial phase in discovering biologically active compounds in a plant involve in the screening of phytochemicals. Phytochemicals are known as secondary metabolites, are bioactive molecules extracted from plants (Saba *et al*. 2023). Analysing phytochemicals from rhizome extracts typically employs both qualitative and quantitative analytical techniques. Different classes of phytochemicals, including phenols, flavonoids, terpenoids, alkaloids and others, each possess unique chemical properties that facilitate identification through specific chemical reagents or analytical methods. The industry can utilise the identification and quantification of these molecules to produce new drugs, cosmetics or food supplements. Using various extraction techniques, both traditional and modern methods, the chemical constituents and biological activity of lempoyang extracts have been investigated in previous studies (Ahmad *et al*. 2023). In previous studies, terpenoids, alkaloids, phenolics and flavonoids were found in the extracts, which have been shown to have anti-inflammatory, antioxidant, antibacterial and many other properties (Haque & Jantan 2017; Kader *et al*. 2011; Ramzan & Zeshan 2023).

Despite numerous studies conducted on the fresh rhizome of lempoyang, there is a literature gap regarding the presented phytochemical groups and the antibacterial activity of the crude extract obtained by subcritical water extraction (SWE). SWE is a relatively new and environmentally friendly extraction method in which water is used under subcritical conditions to extract compounds from plant material. It is a process that utilises the solvent power of water at elevated temperatures (between 100°C and 374°C) while generating a pressure below the supercritical level (usually between 1 bar and 221 bar), which is sufficient to keep the water in liquid form (Asl & Khajenoori 2013; Essien *et al*. 2020). This method has proven successful in extracting bioactive compounds from the dried rhizome of lempoyang (Amir *et al*. 2020; Wahab *et al*. 2022). Given the potential benefits of the dried rhizome extracts of lempoyang, it is also important to investigate the potential of SWE to obtain crude extracts with high bioactivity from the fresh rhizome of lempoyang. The evaluation of crude extracts from SWE could provide new insights into the chemical composition and bioactivity of lempoyang from the fresh rhizome.

Lempoyang extracts have long been used as a remedy for various infections due to their antibacterial properties. These extracts have been shown to be effective against various types of bacteria, including Gram-negative bacteria such as *Escherichia coli* and *Helicobacter pylori*, as well as Gram-positive bacteria such as *Staphylococcus aureus* and *Staphylococcus epidermidis*, with greater efficacy against the latter (Ramzan & Zeshan 2023; Sidahmed *et al*. 2015). Previous studies by Kader *et al*. (2011), Raj *et al*. (2022) and Woo *et al*. (2021) have demonstrated the significant antibacterial activity of fresh rhizome extracts from lempoyang obtained by various extraction methods against various plant pathogenic bacteria. Therefore, this study aims to investigate the phytochemical compounds in the crude extracts of the fresh rhizome of lempoyang obtained by SWE and to evaluate the antibacterial activity of the crude extract against different types of Gram-positive and Gram-negative bacteria.

## MATERIALS AND METHODS

In this study, a comprehensive experimental procedure was developed and carried out, comprising several crucial steps. First, fresh rhizomes of lempoyang were procured and carefully prepared for subsequent processing. SWE was then employed to extract the compounds from the plant material. A qualitative study was conducted through a preliminary screening of phytochemical compounds to identify the chemical groups present in the crude extracts. Subsequently, a gas chromatography-mass spectrometry (GCMS) analysis was performed to quantitatively determine the specific chemical compounds. Finally, the antibacterial activity of the crude extracts was assessed using the Kirby-Bauer diffusion method.

### Acquisition of Lempoyang Rhizomes

The fresh rhizomes of lempoyang were procured from Naturemedic Laboratories Sdn. Bhd., Chendering, Terengganu, Malaysia. The plant was identified and authenticated by the Herbarium Universiti Sultan Zainal Abidin with voucher specimen UniSZA/A/000000006.

### Plant Preparation

**Figure 1:**
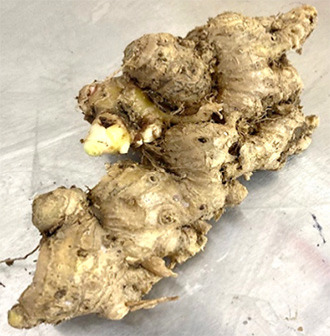

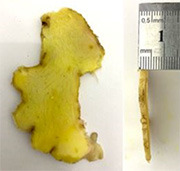
(a) Lempoyang rhizome; (b) Cross-section of lempoyang rhizome.

The fresh rhizomes of lempoyang were examined to remove soil and infested rhizomes. Subsequently, the examined rhizomes were cut into approximately 2 mm to 3 mm thick slices using a cutting machine, as shown in Fig. 1. Following this, the rhizomes were washed under running tap water and rinsed several times to ensure they were free of impurities. Immediately afterwards, all sliced rhizomes were subjected to SWE to maintain their freshness.

### Subcritical Water Extraction (SWE)

The extraction process occurred within an industrial-scale SWE system, specifically in a 70-litre stainless steel batch vessel fabricated by Natxtract, a company of AM Zaideen Ventures Sdn. Bhd. based in Malaysia. To ensure proper cooling, the cooling vessel was pre-cooled to a temperature between 25°C and 27°C by activating the cooling bath (Lauda, Alpha RA 12) before commencing the extraction process began. In summary, 8 kg of cleaned rhizomes were loaded into a stainless-steel mesh basket and immersed in the SWE vessel at a ratio of 1:4 rhizomes to reverse osmosis water (solid:solvent). The stainless-steel lid was then securely closed to engage the motor and agitator shafts for efficient movement and extraction.

Nitrogen gas (99.9%) was introduced into the headspace of the SWE vessel by adjusting a pressure regulator to about 1.5 bar. Any escaping air from the lid of the vessel was checked and immediately rectified by opening the SWE lid again and retightening the nut screw. Nitrogen gas regulated the pressure inside the SWE before the process began. The electric heater was switched on to heat the SWE to the desired extraction temperature. Once the temperature and pressure reached a subcritical state, approximately between 100°C and 110°C and 10 bar to 12 bar, the heater was switched off, but the agitator remained in operation. The extraction time was recorded for 5 min using a stopwatch.

Upon completion of the extraction, the agitator was switched off. The pressure relief valve (PRV) was carefully opened to release the hot air in the SWE vessel until the pressure reached 0 bar. The drain valve of the SWE vessel was opened to allow the hot crude extract stream to flow into a cooling vessel using a centrifugal pump. The extract was cooled to around 40°C to 50°C before collecting the product. The crude extracts were stored in the freezer before undergoing freeze-drying. The SWE and cooling vessels were cleaned, rinsed and allowed to rest before repeating the extraction process with different extraction times: 10 min, 15 min, 20 min and 25 min, while keeping the other variables constant.

### Freeze-Drying Process

After completing SWE, all obtained extracts underwent freeze-drying using a modified version of the published method (Hanafi *et al*. 2021). The crude extracts were evenly distributed on trays and placed in the single product chamber of a pilot-scale freeze dryer (Cuddon FD80, New Zealand). The drying process consisted of three stages: Initial freezing, primary drying and secondary drying. The cooling parameters were set as follows: a freezing temperature of −20°C, a sublimation temperature ranging −20°C to 0°C and a vacuum pressure of 0.34 mbar. The isothermal desorption process occurred in a temperature range of 0°C to 60°C under a vacuum environment of 0.060 mbar. The entire freeze-drying process took approximately 34 h, concluding at a final temperature of 40°C. Subsequently, the freeze-dried extracts were stored in a tightly sealed PU bottle and labelled for further analysis.

### Preliminary Screening of Phytochemical Compounds

Preliminary screening of phytochemical compounds for the presence of various compounds such as phenols, alkaloids, flavonoids, terpenoids, glycosides, steroids, tannins, saponins, anthocyanins, quinones and anthraquinones in the samples was carried out using standard qualitative methods. Methanolic samples were prepared by dissolving 50 mg of each freeze-dried extract in 10 mL of methanol (CH_3_OH), resulting in a sample concentration of 5 mg/mL. However, for the saponin test, the samples were dissolved in distilled water instead of CH_3_OH.

**Figure 2:**
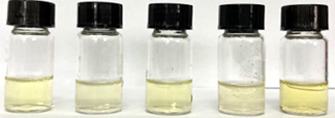
Filtered methanolic crude extract at different extraction times: (a) 5-minute, (b) 10-minute, (c) 15-minute, (d) 20-minute, and (e) 25-minute.

The solutions were shaken and sonicated before being filtered into sample bottles using a nylon syringe filter (diameter: 13 mm, pore size: 0.45 μm). Fig. 2 shows the filtered crude extract of the fresh rhizome of lempoyang which appeared as a transparent solution with cream to pale yellow colour at all extraction times. The phytochemical groups of compounds were tested directly in the samples using different methods, with some adjustments. For each test, 2 mL of each sample was used and performed in triplicate.

#### Test for phenolics

The presence of phenols group in each sample was examined using the ferric chloride (FeCl_3_) test (Akter *et al*. 2023; Gupta *et al*. 2023; Minarni *et al*. 2023). A total of 1 g of FeCl_3_ was dissolved in 20 mL distilled water to prepare a 5% FeCl_3_ solution. The mixture was shaken to obtain a brownish-orange solution. Then, a few drops of the 5% FeCl_3_ solution were added to 2 mL of each sample. A black-purple colour (Minarni *et al*. 2023), blue, violet, purple, green or red-brown spot (Akter *et al*. 2023), or a dark green colour (Gupta *et al*. 2023) in the solution indicate the presence of phenolic groups.

#### Test for alkaloids

The group of alkaloids in each sample was screened using the Wagner’s test. Firstly, the Wagner’s reagent was prepared by dissolving 2 g potassium iodide (KI) and 1.27 g iodine (I) in 20 mL distilled water using a magnetic stirrer. The mixture was then diluted with 80 mL of distilled water to obtain 100 mL of a dark brown, clear solution. Next, 1% (v/v) hydrochloric acid (HCl) was prepared in the fume hood by dissolving 1 mL of HCl in 99 mL of distilled water. A total of 1 mL of the 1% HCl solution was then added to each sample, which was placed in a water bath in the fume hood and rested for 5 min. A few drops of the Wagner’s reagent were then added to the sample solution, resulting in a reddish-brown precipitate indicating the presence of alkaloids (Akter *et al*. 2023; Basumatary 2016).

#### Test for flavonoids

The group of flavonoids in each sample was detected by an alkali reagent test. A 10% (w/v) sodium hydroxide (NaOH) solution was prepared by dissolving 10 g NaOH pellets in 100 mL distilled water. The mixture was mixed with the sonicator to ensure thorough mixing and to accelerate the dissolution of the NaOH pellets in distilled water. The mixture was then shaken to ensure complete dissolution. One millilitre (1 mL) of the 10% NaOH solution was added to each sample and left for a while to obtain a concentrated yellow colour. The disappearance of the colour after the addition of 1 mL of 1% (v/v) HCl indicates the presence of flavonoids (Gul *et al*. 2017; Preshahdin *et al*. 2023).

#### Test for terpenoids

The presence of terpenoids group in each sample was evaluated using the Salkowski test (Hasan *et al*. 2023; Gul *et al*. 2017; Rao *et al*. 2016). The experiment was performed in a fume hood for safety reasons. The total of 2 mL of each sample was mixed with 2 mL of chloroform (CHCl_3_) and shaken well. Then, 2 mL of concentrated sulphuric acid (H_2_SO_4_) was added along the side of the sample bottle until a layer formed. A grey colour (Gul *et al*. 2017) or a reddish-brown colour (Hasan *et al*. 2023; Rao *et al*. 2016) indicates the presence of terpenoid groups.

#### Test for steroids

The group of steroids group in each sample was identified using a previously performed steroid test. For safety reasons, the test was performed in a fume hood. 2 mL of saturated H_2_SO_4_ was carefully introduced along the side of a test tube containing 2 mL of each sample. Then 2 mL of CHCl_3_ was added. A red colouration was observed in the lower chloroform layer, indicating the presence of steroids (Hasan *et al*. 2023; Gul *et al*. 2017).

#### Test for glycoside

The glycoside group in each sample was identified using an aqueous NaOH assay. A few drops of a prepared 10% NaOH solution were added to each sample in separate test tubes. The presence of glycosides was indicated by the appearance of a yellow colour (Basumatary 2016; Rao *et al*. 2016).

#### Test for tannins

The tannin group was evaluated with the Braymer test. A few drops of prepared 5% (w/v) FeCl_3_ were added to each sample. The presence of tannins was confirmed by the development of a black-blue or green-black colouration or the formation of a precipitate. A greenish-black precipitate (Preshahdin *et al*. 2023; Rao *et al*. 2016) or a blue colouration (Basumatary 2016) indicate the presence of tannins. The appearance of a black-blue colour indicated the presence of gallic tannins, and a green-black colour indicated the presence of catechol tannins (Auwal *et al*. 2014).

#### Test for saponins

The group of saponins in each sample was determined by the frothing test. Each sample was vortexed at room temperature. The presence of saponins was detected by the formation of a layer of foam 1 cm thick, which persisted for at least 5 min (Gupta *et al*. 2023; Preshahdin *et al*. 2023).

#### Test for anthocyanins

The group of anthocyanins was investigated using the hydrochloric acid test. The experiment was carried out in a fume hood for safety reasons. A 10% (v/v) ammonia solution was prepared by mixing 10 mL of ammonia with 90 mL of distilled water. Then, 2 mL of each sample was mixed with 2 mL of HCl, which reportedly turned the solution pinkish red. Then, 1 mL of 10% (v/v) ammonia (NH_3_) was added to the solution, which indicated the presence of anthocyanins by forming a violet-blue colour (Obouayeba *et al*. 2021; Saba *et al*. 2023).

### Characterisation of Chemical Compounds

The aim of this analysis was to identify the different chemical compounds in the samples and thus characterise the chemical compound profile of the extracted material. The samples were characterised using a GCMS instrument with a few modifications (Dash *et al*. 2020a; Nik Norulaini *et al*. 2009; Rawat *et al*. 2023; Tian *et al*. 2020). Initially, 2 g of each crude extract was diluted with 2 mL of n-hexane (C_6_H_14_) to achieve a sample concentration of 1 mg/mL. The mixtures were sonicated using an ultrasonic basin (Sukinbo, China) and vigorously shaken using a vortex mixer (Heidolph, Germany) to ensure effective extraction and dissolution. The mixtures were then filtered separately through a nylon syringe membrane (13 mm × 0.45 μm) to remove visible suspended solids prior to analysis. Subsequently, 1 mL of the filtered samples were transferred to vials and placed in the GCMS.

The chemical compounds were identified using an Agilent 7890A GC model (Agilent, Wilmington, USA) equipped with an Agilent 5975C MS model (Agilent, Wilmington, USA). Helium gas was used as the carrier gas with a continuous flow rate of 1 mL/min. The column used was an HP-5MS (30 m × 0.25 mm × 0.25 μm film thickness) silica capillary column. A volume of 1 μL sample was injected into the GCMS in split mode without derivatisation. An electron ionisation energy system was used for detection. The injector temperature, ion source temperature and interface temperature were maintained at 46°C, 190°C and 260°C, respectively. The column temperature was programmed from 46°C for 2 min to 310°C for 8 min at a rate of 15°C/min. Samples were injected by splitting and the splitting ratio was 10:1. Compounds were identified by matching the mass spectral fragmentation pattern of the detected components with the National Institute of Standards and Technology 2017 mass spectral library (NIST 17).

### Assessment of Antibacterial Activity

The antibacterial activity of the sample were assessed against two Gram-positive and four Gram-negative bacteria using the susceptibility Kirby-Bauer disc diffusion method (Asan *et al*. 2022; Bauer *et al*. 1966; Ramzan & Zeshan 2023).

#### Selection of bacterial

Two Gram-positive bacteria: *Bacillus subtilis* (B29), and *Staphylococcus aureus* (ATCC 43300), and four Gram-negative bacteria: *Escherichia coli* (ATCC 25922), *Pseudomonas aeruginosa* (ATCC 15442), *Salmonella choleraesuis* (ATCC 10708) and *Serratia marcencens* (S381) were selected for antibacterial activity assessment. All strains were obtained from the Institute of Bioscience (IBS), Universiti Putra Malaysia, Serdang. The strains were maintained at 40°C on nutrient agar plates throughout the experiment and used as stock cultures.

#### Nutrient agar plate preparation

The total of 34 g of Mueller Hinton Agar (Merck, Germany) powder was accurately weighed and then suspended in 1 L of distilled water in a Schott bottle. The mixture was heated to boiling to ensure complete dissolution of the medium, making it transparent. The Schott bottle containing the mixture was sealed and covered with aluminium foil. It was then sterilised in an autoclave at 121°C for 15 min. In a laminar flow, the sterilised agar was then poured into Petri dishes, which were to have a shallow depth of 4 ± 0.5 mm. The agar plates were waited until the medium solidified and the lid was immediately closed to prevent contamination. The plates were then stored in a sealed container before the bacterial suspension was wiped off.

#### Susceptibility testing by disc diffusion method

The antibacterial activity of the samples was investigated by the disc diffusion method of Bauer *et al*. (1966) in triplicate using 24 h grown strains seeded on nutrient media. Saline water (0.85% NaCl) was added to the cultures to obtain a suspension with a concentration of 1 to 2 × 10^8^ CFU/mL with 0.5 McFarland standard. Approximately 100 μL of the suspension was swirled thoroughly and then spread onto agar plates with a swab to achieve uniform microbial growth. The swab was swabbed three times over the entire surface of the agar, each time at a 60° angle to the previous swab to ensure even distribution of the inoculum.

Sterile filter paper discs (Whatman’s No. 5, 6 mm diameter) were impregnated with 10 μL of each sample (10 mg/mL and 100 mg/mL) and placed on the surface of the plate. All plates were sealed with parafilm to prevent evaporation of the test samples. The plates were then incubated at 37°C for 24 h. The zone of inhibition was determined by measuring the diameter of the clear zone around each disc in mm. The procedure was repeated for a control plate with streptomycin (10 μg/disc) as a positive control.

## RESULTS AND DISCUSSION

### Phytochemical Groups Screening

Table 1 provides an overview of the presence or absence of phytochemical groups in the samples at various extraction times. The presence of phytochemical groups is denoted by (+), while their absence by (−). Phenols, alkaloids, flavonoids, terpenoids, glycosides, tannins and saponins showed presence (+) at all extraction times. In contrast, steroids, and anthocyanins were exhibited absent at all extraction times (−).

The results indicated that the SWE method effectively extracted these groups from the lempoyang rhizomes within a relatively short period. The consistent presence of these compounds at all extraction times could be due to their solubility and stability in the subcritical water environment. However, the absence of certain compounds suggested that they were not effectively extracted under the conditions used in this study or that they were not present, or present only to an insignificant extent in the lempoyang rhizomes.

Initially, all methanolic samples appeared as a transparent solution with a cream to pale yellow colour, as shown in Fig. 1. Methanol was chosen as a solvent for screening phytochemical compounds due to its high extraction yield and strong polarity, facilitating the uptake of a broad range of bioactive compounds (Truong *et al*. 2019). Numerous studies have proven and validated this solvent, highlighting its reliability and suitability for phytochemical screening. Additionally, the screening analyses revealed distinct colour changes within the methanolic samples, serving as indicative markers for the presence of certain chemical groups.

Detailed documentation of these observed colour changes can be found in Table 2. Overall, the consistent presence of phenols, alkaloids, flavonoids, terpenoids, glycosides, tannins and saponins in the samples was aligned with the findings from previous studies utilising the same aqueous extractant. Literature reviews consistently showed the lempoyang extract contained abundant phenols, alkaloids, flavonoids, terpenoids, glycosides, tannins and saponins (Hasan *et al*. 2023; Akter *et al*. 2023; Auwal *et al*. 2014; Basumatary 2016; Gul *et al*. 2017; Gupta *et al*. 2023; Preshahdin *et al*. 2023; Rao *et al*. 2016). Therefore, this study confirmed that SWE is an effective method for extracting and preserving specific active compounds from lempoyang rhizomes consistent with the rich phytochemical composition reported in previous studies.

However, the absence of steroids in the samples is consistent with previous studies conducted with different extraction methods. In studies with an aqueous extract by Hashemi *et al*. (2008) and an ethanolic extract analysis by Aji *et al*. (2022), no steroids were detected either. This consistent finding indicated that the lempoyang rhizomes generally did not contain significant amounts of steroids. However, steroids were found in trace amount (0.63%) once in an acetone extract using a quantitative method (GCMS) (Dash *et al*. 2020b). This suggested that the choice of solvent and analytical method could influence the detection of certain compounds in the extract.

Additionally, previous studies using a solvent extraction method with acetonitrile and 4% acetic acid have consistently documented the absence of anthocyanins in lempoyang rhizomes (Lako *et al*. 2007). Furthermore, it was important to note that the lempoyang rhizomes lacked the physical characteristics associated with the presence of anthocyanins, such as the typical red, purple and blue colouring commonly found in fruits, vegetables or plants known to contain anthocyanins (Abdelmoez *et al*. 2010). This inherent absence of colouration in lempoyang rhizomes is aligned with the absence of anthocyanins and strengthens the understanding of their phytochemical composition.

Overall, variations in extraction time had no significant effect on the qualitative presence or absence of phytochemical groups, as indicated by consistent colour changes or disappearance of colour in the samples regardless of extraction time. However, it was important to note that the main difference between the samples was in the quantitative aspects. Qualitative analyses appeared to be less sensitive and specific compared to quantitative analyses, a factor that could influence the overall results (Tzima *et al*. 2018). Therefore, conducting quantitative studies is important to validate the presence and concentration of phytochemical compounds.

### Chemical Compounds by GCMS

**Figure 3:**
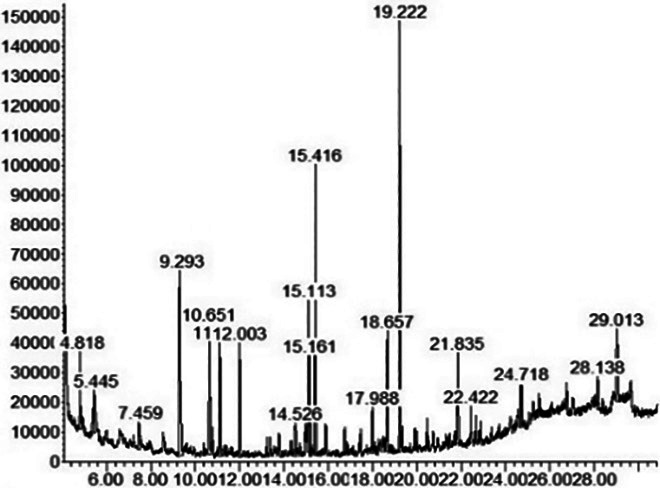

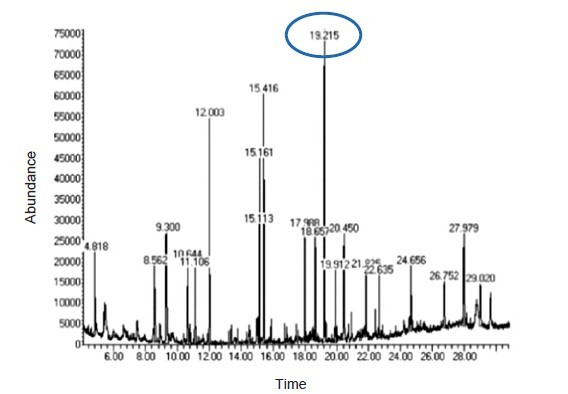

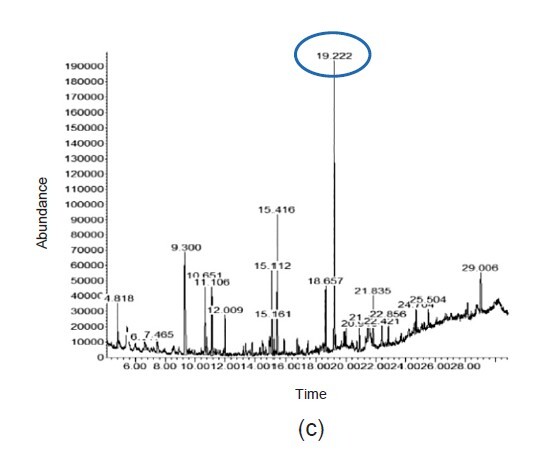

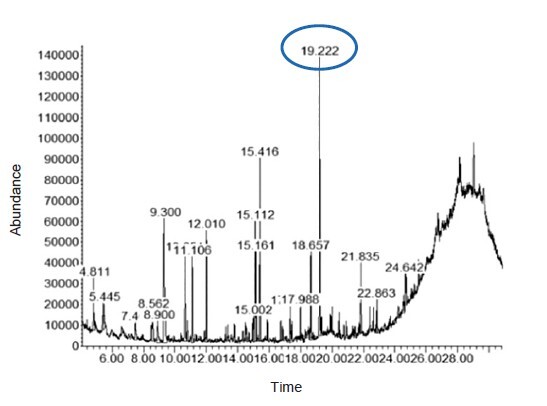

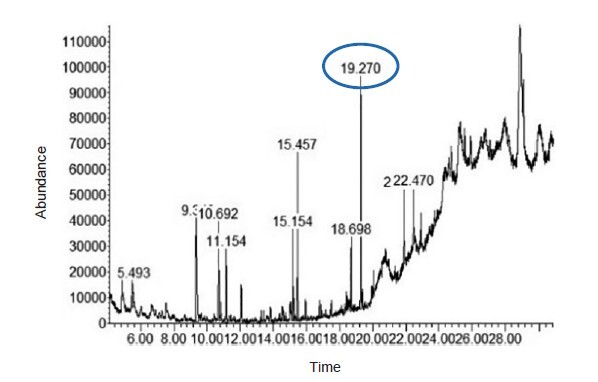
GCMS chromatograms of crude extracts at different extraction times, emphasising the predominant peaks in the 19.215 min to 19.270 min, indicating zerumbone: (a) 5-minute; (b) 10-minute; (c) 15-minute; (d) 20-minute; and (e) 25-minute.

Fig. 3 shows the chromatographic GCMS profile of the samples extracted at different time intervals. The x-axis represents the retention time (minutes), while the y-axis indicates the intensity or abundance of the detected compounds. The chromatogram recorded retention times up to about 29 min and displayed about 20 different chemical compounds represented by individual peaks. Each peak in the chromatogram corresponds to a specific compound in the samples, with the area under each peak proportional to the concentration of the compound.

Notably, in this chromatogram, the highest peak was found in the range of 19.215 min to 19.270 min, identified as zerumbone. This observation emphasised that zerumbone was the predominant compound extracted from the lempoyang samples under the indicated extraction conditions with subcritical water and highlights its importance in the composition of the extracts.

Meanwhile, Table 3 expanded the chromatographic profile by listing the chemical compounds present in the samples compared to NIST17. The table included retention time (minutes), 13 chemical compounds detected in most samples, and their percentage abundance (%) in a sample at different extraction times. The compounds were listed in order of elution from the column and the analysis highlighted the most prominent compound in the extract. The retention time indicated how long each compound took to pass through the chromatographic column, facilitating its separation and characterisation. In addition, the percentage abundance indicated the relative concentration of each compound in the sample at different extraction times.

Zerumbone (2,6,10-Cycloundecatrien-1-one, 2,6,9,9-tetramethyl-, (E,E,E)-), stands out among the 13 compounds identified. It consistently exhibited the highest percentage of peak area at almost all extraction times, ranging from 17.15% to 28.72% at retention times between 19.215 min and 19.270 min with the 15-minute extraction yielding the highest concentration. These results were in agreement with previous studies by Dash *et al*. (2020a), Huong *et al*. (2020) and Sam *et al*. (2019) who extracted zerumbone as the predominant and major component from the lempoyang rhizomes, emphasising the reliability and validity of the current research findings.

Zerumbone amount remained stable despite the fluctuations in the different samples, emphasising its exceptional solubility and strong affinity for subcritical water. However, the fluctuation of zerumbone content across different extraction times can be attributed to the solubility of the solute in SWE due to the properties of the water, the structure of the sample matrix and the solute, and the complex interactions between the water and the solute (Carr *et al*. 2011). These interactions could vary over time and did not always lead to a direct increase in concentration with longer extraction times. Furthermore, like many natural compounds, zerumbone can be susceptible to degradation or chemical changes under certain conditions (Abdelmoez *et al*. 2010).

At the same time, ethanol,1-(2-butoxyethoxy)- was found to be the second most abundant compound with retention times between 9.290 min and 9.307 min and varying percentages of area under the peak between 11.28% and 25.05%. Conversely, compounds such as benzene, 1,3-bis(1,1-dimethylethyl)- with retention times between 10.644 min and 10.692 min showed fluctuation tendencies, indicating their potential volatility and susceptibility to changes during the extraction process. Several other compounds were identified in all crude extracts, albeit at lower levels, such as 2,4-di-tert-butylphenol and octacosane, heneicosane, hexacosane, 1-iodo- and tetracosane.

### Antibacterial Activity

**Figure 4:**
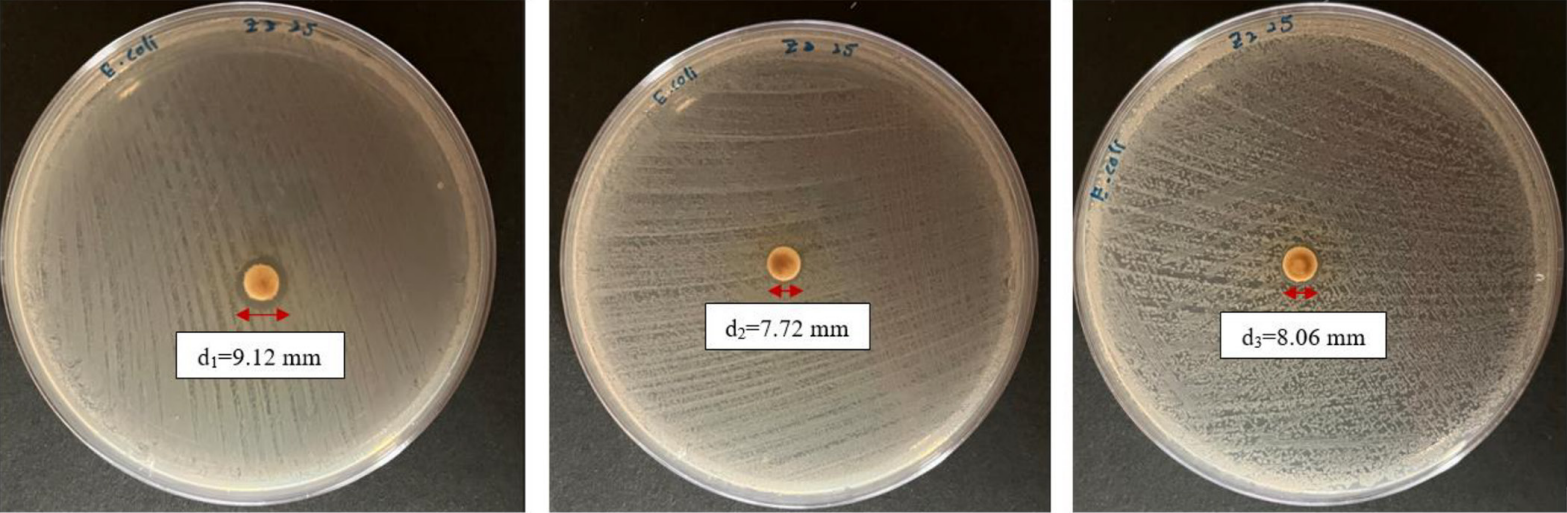
Inhibition zone observations for 100 mg/mL of 25-minute sample against *E. coli*.

Table 4 summarised the antibacterial activity of the samples against Gram-positive and Gram-negative bacteria. Different extraction times and two concentrations (10 mg/mL and 100 mg/mL) were tested, with the diameters of the inhibition zones measured in millimetres (mean ± standard deviation of triplicate tests). The 25-minute sample showed weak inhibitory activity against Gram-negative bacteria, *E. coli* at a concentration of 100 mg/mL with an inhibition zone diameter of 8.63 ± 0.36 mm, while the other samples showed no antibacterial activity. In addition, Fig. 4 illustrated the zones of inhibition of the triplicate sample of 25-minute against *E. coli*. Although the zone of inhibition of the 25-minute sample was weaker compared to the positive control (streptomycin disc, 24.79 ± 0.61 mm), it showed potential as an antibacterial agent.

Gram-positive bacteria were generally more sensitive to antibacterial agents than Gram-negative bacteria, characteristic attributed to the differences in their cell structures. Gram-negative bacteria possess an outer membrane that serves as a barrier, impeding the penetration of antibiotics and other substances to reach their target. Moreover, the presence of efflux pumps in Gram-negative bacteria actively expels antibiotics, which increases their resistance (Epand *et al*. 2016; Jubeh *et al*. 2020; Reygaert 2018).

In contrast, the 25-minute sample exhibited a surprising trend, more effective in inhibiting Gram-negative bacteria than Gram-positive ones. This intriguing result contradicted previous studies that demonstrated higher efficacy of similar samples against Gram-positive bacteria (Ramzan & Zeshan 2023; Sidahmed *et al*. 2015). These findings suggested that the 25-minute sample may possess unique properties or contain specific phytochemicals, allowing it to penetrate the outer membrane of Gram-negative bacteria. It is worth noting that differences in the biochemical processes and physiological properties of bacteria could influence their sensitivity to different phytochemicals (Zhou & Li 2015). Additionally, the concentration of the extract may not have been sufficient to exert an antibacterial effect on Gram-positive bacteria. Further studies were needed to clarify the reasons for the observed differences in antibacterial activity.

Table 5 compares the antibacterial activity of employing extracts (crude extract and essential oil, EO) through aqueous extraction. Previous studies suggested that the EO of the lempoyang fresh rhizome might have been more effective in inhibiting bacterial strains than its crude extract. However, it was important to note that several factors might have influenced the antibacterial properties of the extract, including the extraction method, the polarity of the solvent and the concentration of the crude extract (Chuah *et al*. 2017). In the extraction of secondary metabolites from medicinal plants, the type and number of compounds extracted might have been influenced by the solvents used. It was also observed that the polarity of the solvent and the duration of the extraction process significantly affect the recovery of bioactive chemicals from plant materials (Malik *et al*. 2019).

Overall, this current study has shown that the crude extract obtained from the fresh rhizome of lempoyang by the SWE extraction method has limited antibacterial activity against the tested bacteria. Although a weak inhibitory effect against *E. coli* was observed at the highest concentration and the longest extraction time, further studies are required to validate these findings and assess the potential of the SWE-extracted lempoyang rhizome against a broad spectrum of bacterial strains. This investigation may contribute to the development of natural antibacterial agents derived from employing rhizome.

### Relationship of Phytochemical Compounds with Antibacterial Activity

Previous studies have highlighted the presence of bioactive compounds such as terpenoids, phenols and flavonoids in lempoyang, which are known for their antibacterial properties (Ashour *et al*. 2018). In addition, zerumbone, a terpenoid compound in lempoyang, has been reported to exhibit antibacterial activity against S*. aureus, B. subtilis, E. coli* and *P. vulgaris*, with the highest percentage of area under the peak ranging from 15.09% to 29.53% at almost all extraction times (Liu *et al*. 2017; Moreira *et al*. 2018).

In this study, the observed antibacterial activity of 25-minute sample likely resulted from the presence of bioactive compounds, including terpenoids, phenols and flavonoids. However, this antibacterial activity seems to be less pronounced compared to findings in previous studies. Several factors, including plant origin, location, extraction techniques and drying methods, can influence the phytochemical composition of plant materials (Ibáñez *et al*. 2023). Therefore, these results emphasise the need for comprehensive testing beyond initial phytochemical screening to thoroughly evaluate the antibacterial potential of the sample for specific applications.

## CONCLUSION

In this study, a lempoyang extract from fresh rhizome was successfully obtained by extraction with subcritical water, revealing the presence of 13 chemical compounds. Zerumbone, a terpenoid, was the predominant compound with percentages ranging from 17.15% to 28.72% at different extraction times, peaking at 15-minute. The samples also contained phenols, alkaloids, flavonoids, saponins and tannins, but no steroids and anthocyanins. Although almost all the extracts exhibited no antibacterial activity against various bacteria, the 25-minute extract at a concentration of 100 mg/mL demonstrated potential antibacterial activity against *E. coli*.

Identification of the chemical compounds by GCMS analysis is important to understand the composition of the extract and its possible role in inhibiting bacterial growth, which offers prospects for new antibacterial agents. To verify the presence and concentrations of specific compounds, additional quantitative analyses using more sensitive and targeted techniques are required. Such analyses would allow a more accurate assessment of the therapeutic potential of the sample and its suitability for specific applications.

## Figures and Tables

**Table 1:** Phytochemical groups in crude extracts.

Phytochemical	5-minute	10-minute	15-minute	20-minute	25-minute
Phenolics	+	+	+	+	+
Alkaloids	+	+	+	+	+
Flavonoids	+	+	+	+	+
Terpenoids	+	+	+	+	+
Glycoside	+	+	+	+	+
Tannins	+	+	+	+	+
Saponins	+	+	+	+	+
Steroid	−	−	−	−	−
Anthocyanins	−	−	−	−	−

*Notes*: (+) = Present; (−) = Absent

**Table 2:** Phytochemical groups screening of samples at different extraction times (Sample from left to right: 5-minute, 10-minute, 15-minute, 20-minute and 25-minute).

Test	Observation	Finding
Phenolics	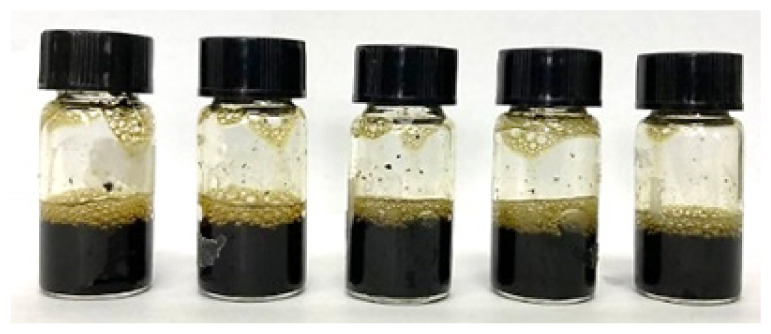	Dark green colour in the solution indicated the presence of phenolic groups (Gupta *et al*. 2023).
Alkaloids	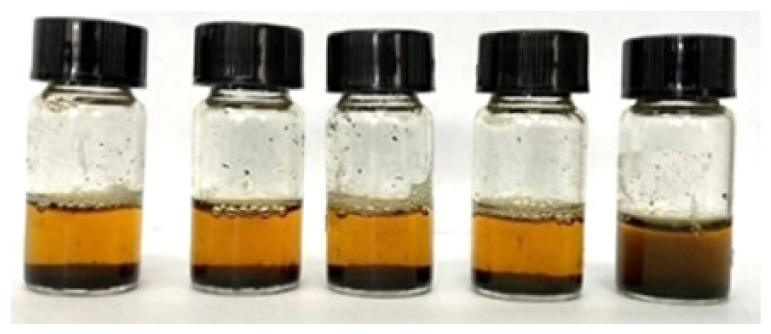	Reddish-brown precipitate indicated the presence of alkaloids (Akter *et al*. 2023; Basumatary 2016).
Flavonoids	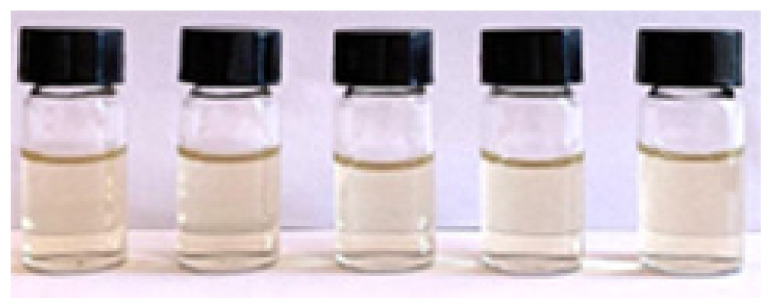	Disappearance or faded of the colour after the addition of 1 mL of 1% (v/v) HCl indicated the presence of flavonoids (Gul *et al*. 2017; Preshahdin *et al*. 2023).
Terpenoids	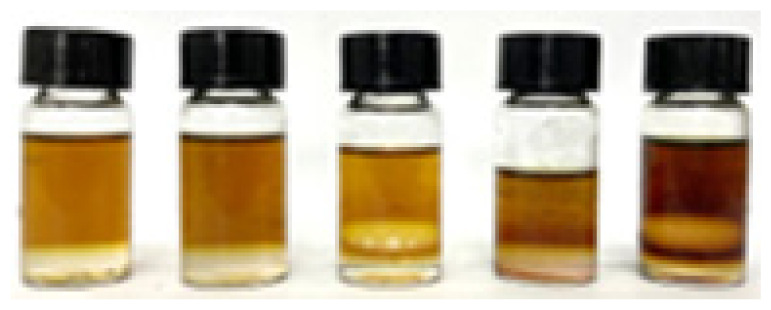	Layer formed and a reddish-brown colour indicated the presence of terpenoid groups. (Hasan *et al*. 2023; Rao *et al*. 2016)
Glycoside	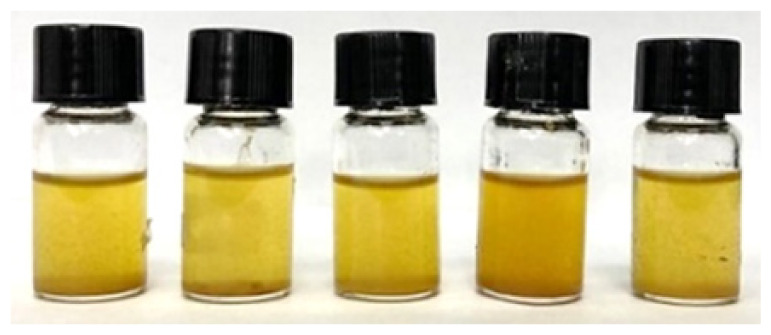	Yellow colour indicated the presence of a yellow colour (Basumatary 2016; Rao *et al*. 2016).
Tannins	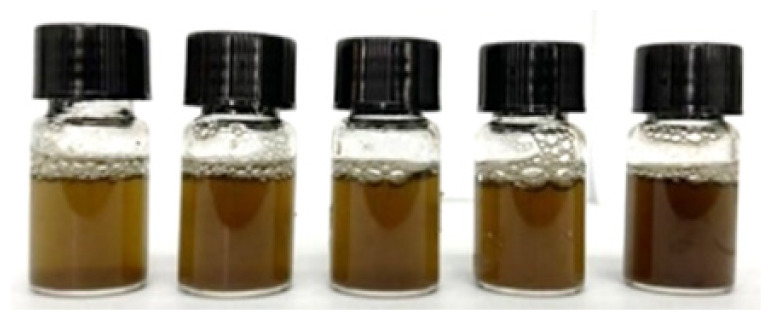	Green-black colour indicated the presence of catechol tannins (Auwal *et al*. 2014).
Saponins	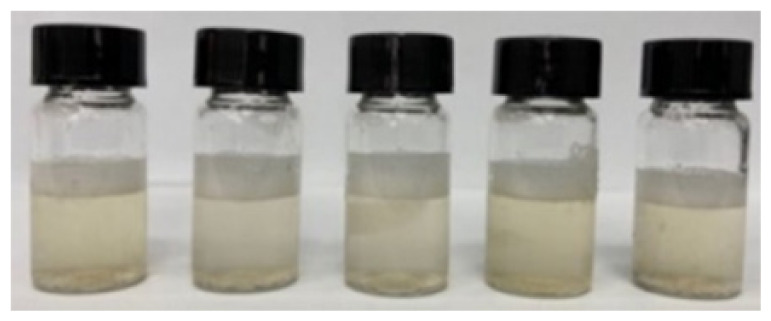	Layer of 1 cm thick foam which persisted for at least 5 min indicated the presence of saponins (Gupta *et al*. 2023; Preshahdin *et al*. 2023).
Steroids	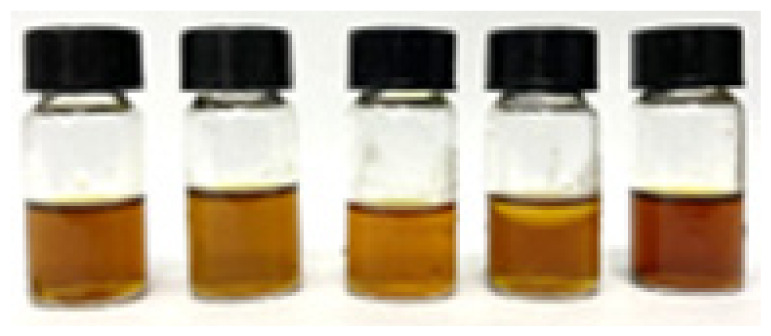	No red colouration was observed in the lower chloroform layer; hence steroids was absent.
Anthocyanins	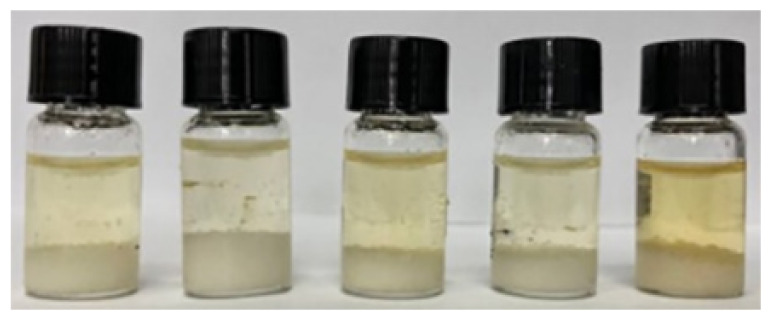	No violet-blue colouration was observed in the lower chloroform layer; hence anthocyanins was absent.

**Table 3:** The chemical compounds in crude extract by comparing with NIST17.

Retention time (minutes)	Chemical compound detected	Abundance (%)				

		Sample (extraction time, minutes)				

		5	10	15	20	25
4.811–4.825	Oxalic acid, cyclohexyl decyl ester	3.97	4.35	3.97	3.88	–
5.445–5.452	Decane	6.89	–	–	3.88	6.11
8.555–8.562	Cyclopentasiloxane, decamethyl-	–	6.75	–	4.13	–
9.293–9.307	Ethanol, 1-(2-butoxyethoxy)-	16.52	11.28	18.55	16.23	25.05
10.644–10.692	Benzene, 1,3-bis (1,1-dimethylethyl)-	9.06	5.91	10.12	8.20	14.65
11.106	Pentacosane	6.51	–	7.36	6.59	–
12.003–12.010	Cyclohexasiloxane, dodecamethyl-	6.14	11.98	4.81	8.27	–
15.161	Cycloheptasiloxane, tetradecamethyl-	4.46	8.73	3.24	5.60	–
15.416–15.457	2,4-Di-tert-butylphenol	12.61	11.90	11.74	10.91	18.08
17.988	Cyclooctasiloxane, hexadecamethyl-	2.10	5.33	–	2.13	–
18.657–18.698	Octacosane^a^ / Heneicosane^b^ / Hexacosane, 1-iodo-c / Tetracosane^d^	5.80^a^	5.24^a^	6.27^b^	5.99^c^	9.05^d^
**19.215–19.270**	**2,6,10-Cycloundecatrien-1-one, 2,6,9,9-tetramethyl-, (E,E,E)-**	**21.06**	**17.15**	**28.72**	**19.41**	**27.06**
21.835	Octacosane^a^ / Heptacosane^b^ / Heneicosane^c^ / Hexadecane, 2-methyl^d^	4.89^a^	3.78^b^	5.21^c^	4.77^d^	–

*Notes*: (–) = Not detected; a, b, c and d represent individual chemical compounds detected at the same retention time (in minutes).

a = Octacosane;

b = Heneicosane;

c = Hexacosane, 1-iodo-;

d = Tetracosane.

The bold data refers to Zerumbone (2,6,10-Cycloundecatrien-1-one, 2,6,9,9-tetramethyl-, (E,E,E)-), which stands out among the 13 compounds identified. This distinction highlights the prominence or significance of Zerumbone in the analysis.

**Table 4:** Antibacterial activity of lempoyang rhizome.

	Inhibition zones (mm)									

Crude extract concentration	10 mg/mL					100 mg/mL				

Microbes	Extraction times (minutes)									

	5	10	15	20	25	5	10	15	20	25
Gram-positive										
*Bacillus subtilis*	–	–	–	–	–	–	–	–	–	na
*Staphylococcus aureus*	na	na	na	na	na	na	na	na	na	na
Gram-negative										
*Escherichia coli*	na	na	na	na	na	na	na	na	na	8.63 ± 0.36
*Salmonella choleraesuis*	–	–	–	–	–	–	–	–	–	na
*Pseudomonas aeruginosa*	–	–	–	–	–	–	–	–	–	na
*Serratia marcencens*	–	–	–	–	–	–	–	–	–	na

*Note*: (-) : not tested; (na) : no activity; diameter of the inhibition zones (mm) including the disc diameter (6 mm) were given as the mean ± SD of triplicate experiments; inhibition zone (mm): weak activity = 7 mm–10 mm, intermediate activity = 11 mm–15 mm, strong activity = ≥ 16 mm (Asan *et al*. 2022).

**Table 5:** Antibacterial activity of lempoyang fresh rhizome extracts through aqueous extraction.

Reference	Extraction method	Product	Inhibition zone diameter (mm)			

			Gram-positive		Gram-negative	
	
			*B. subtilis*	*S. aureus*	*E. coli*	*P. aeruginosa*
Current study	SWE	Crude	na	na	8.63 ± 0.36	na
Preshahdin *et al*. (2023)	Hydrodistillation	EO	–	13.33 ± 0.58	16.66 ± 0.58	–
Tian *et al*. (2020)	Hydrodistillation	EO	9.99 ± 1.86	14.54 ± 3.78	9.36 ± 1.98	8.34 ± 0.70
Padalia *et al*. (2018)	Hydrodistillation	EO	na	10 ± 1.00	6 ± 1.00	na
Azelan *et al*. (2015)	Hydrodistillation	EO	–	13 ± 1.00	12 ± 0.00	11.3 ± 0.50
Helen *et al*. (2009)	Hydrodistillation	EO	–	12 ± 0.00	–	–
Ungsurungsie *et al*. (1982)	Maceration	Crude	2 ± 0.00	–	–	–

*Note*: EO: Essential oil; (-) : not tested; (na) : no activity; given as the mean ± SD of triplicate experiments; inhibition zone (mm): weak activity = 7 mm–10 mm, intermediate activity = 11 mm–15 mm, strong activity = ≥ 16 mm (Asan *et al*. 2022).
